# Association of cigarette smoking with cardiometabolic risk factors: A cross-sectional study

**DOI:** 10.18332/tid/191246

**Published:** 2024-07-26

**Authors:** Samar Sultan, Fouzy Lesloom

**Affiliations:** 1 Medical Laboratory Sciences, Faculty of Applied Medical Sciences, King Abdulaziz University, Jeddah, Saudi Arabia; 2 Regenerative Medicine Unit, King Fahad Medical Research Centre, King Abdulaziz University, Jeddah, Saudi Arabia

**Keywords:** smoking, cardiometabolic risk factors, liver function tests, high-sensitivity cardiac troponin I

## Abstract

**INTRODUCTION:**

Despite strong and consistent epidemiological evidence linking cigarette smoking to several cardiovascular diseases (CVDs), the association between smoking intensity and CVD risk factors remains unclear. This study aimed to explore the possible effects of cigarette smoking on cardiometabolic risk in healthy individuals.

**METHODS:**

This cross-sectional study was conducted between November 2022 and June 2023. Consecutive sampling was performed to include 160 healthy participants: 100 smokers with 60 males and 40 females; and 60 age- and sex-matched non-smokers with 36 males and 24 females. Blood samples were taken from each participant to assess their cardiometabolic function: lipid profile, von Willebrand factor (vWF), high-sensitivity cardiac troponin I (hs-cTnI), and fibrinogen levels; and liver function using an automated enzymatic method. In addition, blood sugar level, body mass index (BMI), and blood pressure were recorded.

**RESULTS:**

Smokers had significantly higher vWF functional activity and hs-cTnI but significantly lower albumin and total bilirubin levels than non-smokers (65.87 ± 19.07 vs 56.45 ± 6.59, respectively, p<0.001; 0.0382 ± 0.0077 vs 0.0147 ± 0.0105, respectively, p<0.001; and 4.63 ± 0.32 vs 4.74 ± 0.28, respectively, p=0.026). The number of cigarettes consumed daily was associated positively and significantly with plasma levels of low-density lipoprotein cholesterol, high-density lipoprotein cholesterol, vWF functional activity, and hs-cTnI but were negatively associated with total bilirubin. Moreover, heavy smokers had a significantly higher BMI and waist-to-hip ratio among male smokers than non-smokers.

**CONCLUSIONS:**

Cigarette smoking was associated with increased dyslipidemia, BMI, and central obesity, in addition to higher vWF functional activity. Altogether, increased hs-cTnI levels in smokers indicate a higher susceptibility to CVD.

## INTRODUCTION

Smoking is a leading health concern^1^, with a heavy toll on life, directly through active inhalation and indirectly via passive inhalation^2^. World ‘No-Tobacco Day’, 31 May, is a reminder of its hazards and the importance of quitting smoking^3^. The tobacco epidemic is considered one of the worst global public health problems, killing more than seven million people annually, with almost six million deaths among smokers due to direct tobacco use, in addition to approximately one million non-smokers who die from exposure to secondhand smoke^4^. Despite great efforts by the Saudi government to fight tobacco smoking, its prevalence continues to grow^5^. A recent study showed that between 2009 and 2015, the prevalence of tobacco smoking in the Saudi population substantially increased from 8.9% to 19.5%^6^. Lighting a cigarette is responsible for several harmful adverse effects on most body organs, including the lungs, heart, and organs, without direct contact with smoke^7^.

The liver is a vital organ affected by smoking, which interferes with its ability to process and expel drugs and toxins^8^. Abnormal liver function test results are associated with a high risk of cardiovascular disease (CVD)^9^. The liver is essential for lipid metabolism, including synthesis, storage, and elimination. Dyslipidemia can be caused by impaired liver function^10^. A disrupted lipid profile characterized by increased levels of low-density lipoprotein (LDL), total cholesterol, and triglycerides, and decreased levels of high-density lipoprotein (HDL) plays a major role in the pathogenesis of CVD^11^. When LDL levels are excessive, cholesterol-rich plaques can form, leading to the constriction and hardening of the blood arteries, a condition known as atherosclerosis^12^. Cigarette smoking can increase triglyceride levels, which are associated with an increased risk of CVD^13^.

Smoking also affects the von Willebrand factor (vWF), a glycoprotein that promotes platelet aggregation and adherence to the wounded vascular wall subendothelium^14^. It is also implicated in hemostasis^15^. Increased vWF is a risk factor for CVD^16^. Furthermore, endothelial dysfunction, a characteristic of atherosclerosis and other CVDs, is indicated by elevated vWF levels^17^.

Fibrinogen is a prothrombotic and inflammatory marker that is elevated in smokers^18^. Cigarette smoking stimulates circulating fibrinogen levels, an independent risk factor for CVD, in a dose-dependent manner^19^. High fibrinogen levels affect blood viscosity, platelet aggregation, and fibrin formation^20^, which are positively associated with atherothrombotic events in smokers^21^.

High-sensitivity cardiac troponin I (hs-cTnI) is a biomarker used to diagnose acute myocardial infarction (AMI), also known as a heart attack. Its assays allow the detection of considerably low cardiac troponin concentrations in healthy and asymptomatic individuals. It also serves as a potential tool for cardiovascular risk stratification in the general population^22^. It is a protein in cardiac muscle cells released into the bloodstream when the cardiac muscle is damaged. Thus, elevated hs-cTnI levels indicate myocardial damage or necrosis, often caused by a disturbance in the blood supply of the heart due to atherosclerotic plaque rupture and subsequent blood clot formation^23,24^. Recent studies have shown that cigarette smokers have lower circulating hs-cTnI^24^.

Therefore, this study aimed to investigate the potential effects of cigarette smoking on cardiometabolic risk factors (lipid profile, vWF, hs-cTnI, liver function, fasting blood glucose, and fibrinogen levels) in 160 healthy individuals.

## METHODS

### Study population and definitions

The study design was cross-sectional, and the study population included those attending primary healthcare centers at East Jeddah Hospital, Jeddah City, Saudi Arabia, for vaccination or those with children visiting well-baby clinics. The study was conducted between November 2022 and June 2023. This study was approved by National Committee of Bioethics, King Abdulaziz City for Science and Technology (reference no: H-O2-3J-OO2). All potential participants were informed of the study’s objectives, and written informed consent was obtained from all subjects and their legal guardians. The participants ages ranged from 25–60 years. Participants were divided into active smokers and non-smokers. Active smokers were defined as those who smoked at least one cigarette per day. Detailed information regarding the number of packs consumed daily by each smoker was recorded. Heavy smokers were defined as those who smoked more than one pack of cigarettes daily.

Participants with a history of CVD or any other significant medical condition; pregnant or lactating women; a history of liver disease, abnormal liver function, or a history of renal disease with the use of medications that could affect the study parameters; a history of smoking cessation within the past year; shisha smokers; and users of electronic or e-cigarettes were excluded. The sample size was calculated to be 130 according to the OpenEpi sample size calculator website, with a two-sided significance level (1-α) of 0.95, 0.8 power, a ratio of exposed/unexposed of 0.6, and an assumed odds ratio of 3. However, the sample size was increased to 160 healthy individuals. Following consecutive sampling, 100 smokers and 60 age- and sex-matched non-smokers were enrolled in the present study. All participants completed a questionnaire regarding their medical history and smoking patterns (Supplementary file Part A). We used a questionnaire adapted from the European Risk Assessment Tool. It is a valid tool that provides acceptable discrimination and good calibration for the risk prediction of chronic cardiometabolic disorders in short- and long-term follow-ups. The predictors included age, body mass index, waist circumference, use of antihypertensive medications, current smoking status, and family history of CVD and/or diabetes. For external validation of the model in the Tehran Lipid and Glucose Study (TLGS), the area under the curve (AUC) and the Hosmer–Lemeshow (HL) goodness-of-fit test was performed for discrimination and calibration, respectively^25^. It consisted of three sections: 1) personal characteristics; 2) smoking patterns; and 3) cardiometabolic risk assessments.

Blood pressure was measured using a validated device after patients were allowed to rest for 15 min. The test was repeated twice with a duration of 10 min between them. Subsequently, the mean of the recorded measurements was calculated for each patient.

### Blood sampling procedures and biochemical assays

Venous blood samples (9 mL) were collected from 100 fasting smokers and 60 non-smokers. The samples were split into three tubes containing 4 mL of lithium heparin, 3 mL of serum, and 2 mL of trisodium citrate, respectively. Samples from the lithium heparin tubes (green top) were analyzed for alanine transaminase (ALT), albumin, total bilirubin, lipid profile, and fasting blood glucose levels. The serum tube (red top) and trisodium citrate samples (blue top) were used to examine hs-cTnI and vWF levels, respectively. Using a Heraeus Labofug centrifuge (Thermo Scientific™, PA, USA), all green- and red-capped tubes were centrifuged once at 4400g for 5 min at room temperature, whereas blue-capped tubes were centrifuged twice at 2000g for 15 min.

After the enzymatic reaction, Alinity C (Abbott, Wiesbaden, Germany) was used to spectrophotometrically evaluate liver function, lipid profile, and fasting blood glucose levels by measuring light absorption. Hs-cTnI levels were measured using Alinity I (Abbott, Wiesbaden, Germany). This two-step immunoassay uses chemiluminescent microparticle immunoassay (CMIA) technology to measure cardiac troponin levels in human serum. The concentrations of vWF were assessed using STA R Max3 (Stago, Parsippany-USA), which assesses clotting time in the presence of cephalin and an activator.

### Statistical analysis

Statistical Package for Social Sciences (IBM SPSS, version 25) was used for data analysis. Descriptive statistics were used to summarize the two compared groups’ demographic, clinical, and laboratory findings. Qualitative variables were expressed as frequencies and percentages, while numerical continuous variables were expressed as means and standard deviations. The normality of quantitative variables was assessed using the Kolmogorov–Smirnov and Shapiro–Wilk tests. A power of 0.8 was chosen for the study, and the effect size (d) was calculated. The minimum sample size was determined using the Raosoft online sample size calculator with a 0.1 margin of error, a 95% confidence level, and a reported prevalence of cardiometabolic risk of 39.8% in the Saudi population^26^. The chi-squared and Student’s t-tests were used to compare qualitative and continuous variables, respectively. Pearson’s correlation coefficient (r) was used to assess the strength and direction of linear relationships. Binary logistic regression analysis was used to identify significant variables related to obesity or being overweight. Crude and adjusted odds ratios (ORs) were calculated with 95% confidence intervals. All tests were two-tailed, and statistical significance was set at p<0.05.

## RESULTS

### Participants

Table 1 shows that participants’ age and sex did not differ significantly according to their smoking status. However, the participants’ body mass index (BMI) differed significantly according to their smoking status, with significantly more obese smokers than non-smokers (21% and 8.3%, respectively; p=0.017). Our results showed that 76% of smokers smoked one pack daily (up to 20 cigarettes per day), whereas 24% smoked more than one pack daily (>20 cigarettes per day).

**Table 1:** Characteristics according to smoking status among healthy people aged 25–60 years, attending primary healthcare centers for vaccination or for visiting well-baby clinics from November 2022 to June 2023, at East Jeddah Hospital, Jeddah City, Saudi Arabia (N=160)

*Characteristics*	*Non-smokers (N=60)*		*Smokers (N=100)*		*p*
	*n*	*%*	*n*	*%*	
**Age** (years)					
<40	42	70.0	64	64.0	
≥40	18	30.0	36	36.0	0.492
**Gender**					
Male	36	60.0	60	60.0	
Female	24	40.0	40	40.0	1.000
**Body mass index** (kg/m^2^)					
Normal <25	8	13.3	4	4.0	
Overweight 25–29.9	47	78.3	75	75.0	0.017^†^
Obese ≥30	5	8.3	21	21.0	

† Chi-squared test (statistically significant at p<0.05).

### The effect of smoking on cardiometabolic tests

As shown in Table 2, smokers had higher lipid profile parameters than non-smokers, with higher serum total cholesterol, LDL cholesterol, and non-HDL cholesterol levels but lower serum HDL cholesterol levels. However, the lipid profile parameter differences between smokers and non-smokers were not statistically significant. Regarding liver function, smokers had significantly lower serum albumin levels (4.63 ± 0.32 and 4.74 ± 0.28, respectively; p=0.026) and significantly lower total mean serum bilirubin levels (0.92 ± 0.05 and 0.96 ± 0.05, respectively; p<0.001), than non-smokers. Regarding hematological findings, smokers had significantly higher vWF functional activity (p<0.001) than non-smokers. Fasting blood glucose levels did not differ significantly according to the smoking status. The hs-cTnI levels were significantly higher in smokers than in non-smokers (p<0.001). Moreover, smokers had significantly higher systolic and diastolic blood pressures than non-smokers (p=0.003 and p=0.004, respectively). Smokers also had a significantly higher waist-to-hip ratio than non-smokers (p<0.001 for males and females).

**Table 2:** Results of cardiometabolic tests according to smoking status among healthy people aged 25–60 years, attending primary healthcare centers for vaccination or for visiting well-baby clinics from November 2022 to June 2023, at East Jeddah Hospital, Jeddah City, Saudi Arabia (N=160)

*Parameters*	*Non-smokers (N=60)*		*Smokers (N=100)*		
	*Mean*	*SD*	*Mean*	*SD*	*p*
Triglycerides	147.98	10.02	147.57	13.89	0.841
Total cholesterol	198.95	11.90	199.40	14.11	0.836
LDL cholesterol	113.58	17.40	116.07	17.35	0.382
HDL cholesterol	48.73	6.01	50.87	8.24	0.082
Non-HDL cholesterol	120.23	16.61	123.73	18.92	0.238
ALT	29.62	2.23	29.69	2.14	0.835
Albumin	4.74	0.28	4.63	0.32	0.026^†^
Total bilirubin	0.96	0.05	0.92	0.05	<0.001^†^
Fasting blood glucose	89.10	5.99	89.56	5.85	0.634
Fibrinogen level	3.51	0.54	3.59	0.86	0.498
vWF functional activity	56.45	6.59	65.87	19.07	<0.001^†^
Cardiac troponin I (ng/mL)	0.02	0.01	0.04	0.01	<0.001^†^
Systolic blood pressure	130.8	9.2	135.5	9.8	0.003^†^
Diastolic blood pressure	91.6	6.4	94.8	7.0	0.004^†^
**Waist-to-hip ratio**					
Males	0.90	0.10	1.00	0.12	<0.001^†^
Females	0.82	0.07	0.90	0.07	<0.001^†^

† Independent samples t-test (statistically significant at p<0.05).

Table 3 shows that the number of cigarettes smoked per day was correlated positively and significantly with participants’ plasma LDL cholesterol (r=0.225, p=0.004), plasma non-HDL cholesterol (r=0.22, p=0.005), vWF functional activity (r=0.41, p<0.001), cardiac hs-cTnI serum level (r=0.69, p<0.001), systolic blood pressure (r=0.30, p<0.001), diastolic blood pressure (r=0.30, p<0.001), BMI (r=0.45, p<0.001), and waist-to-hip ratio (r=0.49, p<0.001) (n=160 participants). Moreover, the number of cigarettes smoked per day was negatively and significantly correlated with HDL cholesterol (r= -0.18, p=0.025) and total bilirubin (r= -0.46, p<0.001) levels. However, the other cardiometabolic function test results were not significantly associated with the number of cigarettes consumed daily.

**Table 3:** Pearson’s correlation coefficient (r) between the number of smoked cigarettes per day and cardiometabolic tests of healthy smokers and non-smokers aged 25–60 years, attending primary healthcare centers for vaccination or for visiting well-baby clinics from November 2022 to June 2023, at East Jeddah Hospital, Jeddah City, Saudi Arabia (N=160)

*Variables*	*Correlation coefficient*	*p*
Triglycerides	0.06	0.456
Total cholesterol	0.15	0.061
LDL-cholesterol	0.23	0.004^†^
HDL-cholesterol	-0.18	0.025^†^
Non-HDL cholesterol	0.22	0.005^†^
ALT	0.13	0.103
Albumin	-0.1	0.216
Total bilirubin	-0.46	<0.001^†^
Fasting blood glucose	0.09	0.257
Fibrinogen	0.12	0.142
vWF functional activity	0.41	<0.001^†^
Cardiac troponin I (ng/mL)	0.69	<0.001^†^
Systolic blood pressure	0.30	<0.001^†^
Diastolic blood pressure	0.30	<0.001^†^
Body mass index	0.45	<0.001^†^
Waist-to-hip ratio	0.49	<0.001^†^

† Statistically significant at p<0.05.

As shown in Table 4, smokers who had smoked for >10 years had significantly higher serum total cholesterol (p=0.023), LDL cholesterol (p=0.011), non-HDL cholesterol (p=0.008), and triglyceride (p=0.031) levels compared with those who had smoked for ≤10 years. Regarding liver function and hematological findings, participants who smoked for >10 years had significantly higher vWF functional activity (p<0.001) than those who smoked for ≤10 years. Moreover, as shown in Table 5, men who smoked for >10 years had a significantly higher BMI (p=0.001), systolic blood pressure (p=0.011), diastolic blood pressure (p=0.011), and waist-to-hip ratio (p=0.003). Other laboratory parameters (triglycerides, LDL cholesterol, non-HDL cholesterol, ALT, total bilirubin, vWF functional activity, and hs-cTnI) did not differ significantly according to the duration of smoking.

**Table 4:** Characteristics according to smoking intensity (pack/day) among healthy smokers aged 25–60 years, attending primary healthcare centers for vaccination or for visiting well-baby clinics from November 2022 to June 2023, East Jeddah Hospital, Jeddah City, Saudi Arabia (N=100)

*Characteristics*	*≤1 pack/day (N=76)*		*>1 pack/day (N=24)*		*p*
	*n*	*%*	*n*	*%*	
**Age** (years)					
<40	51	79.7	13	20.3	
≥40	25	69.4	11	30.6	0.330
**Gender**					
Male	41	68.3	19	31.7	
Female	35	87.5	5	12.5	0.028^†^
**Body mass index** (kg/m^2^)					
Normal <25	3	75.0	1	25.0	
Overweight 25–29.9	73		23	14.7	<0.001^†^
Obese ≥30	9	42.9	12	57.1	

† Chi-squared test (statistically significant at p<0.05).

**Table 5:** Results of laboratory findings according to the duration of smoking among healthy smokers aged 25–60 years, attending primary healthcare centers for vaccination or for visiting well-baby clinics from November 2022 to June 2023, at East Jeddah Hospital, Jeddah City, Saudi Arabia (N=100)

*Parameters*	*≤10 years (N=61)*		*>10 years (N=39)*		
	*Mean*	*SD*	*Mean*	*SD*	*p*
Triglycerides	149.25	12.43	142.25	16.96	0.031^†^
Total cholesterol	197.61	12.48	205.08	17.47	0.023^†^
LDL-cholesterol	113.61	17.33	123.88	15.29	0.011^†^
HDL-cholesterol	50.05	7.36	53.46	10.31	0.077
Non-HDL cholesterol	120.93	16.05	132.58	24.35	0.008^†^
ALT	29.50	2.22	30.29	1.76	0.115
Albumin	4.07	0.19	4.11	0.21	0.359
Total bilirubin	0.92	0.04	0.89	0.05	0.003^†^
Fasting blood glucose	89.07	5.36	91.13	7.07	0.133
Fibrinogen level	3.53	0.52	3.78	1.51	0.230
vWF functional activity	60.91	9.49	81.58	30.55	<0.001^†^
Cardiac troponin I (ng/mL)	0.038	0.01	0.039	0.01	0.484
BMI	28.03	1.70	29.53	2.39	0.001^†^
Systolic blood pressure	134.08	9.24	139.83	10.41	0.011^†^
Diastolic blood pressure	93.86	6.46	97.88	7.29	0.021^†^
**Waist-to-hip ratio**					
Males	0.96	0.10	1.05	0.13	0.003^†^
Females	0.90	0.07	0.89	0.07	0.720

† Independent variable t-test (statistically significant at p<0.05).

As shown in Supplementary file Table S1, smokers who smoked >1 pack/day had higher lipid profile parameters, with significantly higher serum total cholesterol (p=0.014), LDL cholesterol (p=0.011), and non-HDL levels (p=0.017) than the other participants. Regarding liver function, participants who smoked >1 pack/day had significantly lower serum albumin levels (p=0.021). Hematological findings revealed that participants who smoked >1 pack/day had significantly higher vWF functional activity (p<0.001) compared to other participants. Fasting blood glucose, levels were also higher among those who smoked >1 pack/day than among those who smoked fewer cigarettes; however, the difference was not statistically significant. The hs-cTnI levels were higher among those who smoked >1 pack/day than among those who smoked ≤1 pack/day. However, the difference in mean troponin I levels was not statistically significant. Systolic and diastolic blood pressures were significantly higher among those who smoked >1 pack/day (both p=0.004). Moreover, the waist-to-hip ratio was significantly higher in men who smoked >1 pack/day (p=0.020).

Supplementary file Table S2 shows the crude odds ratio (OR) and adjusted odds ratio (AOR) as indicated by the binary logistic regression analysis of the predictor variables for obesity and underweight state among all the participants (n=160). Significant predictors were fasting blood glucose (OR=0.16; 95% CI: 0.09–0.3, and AOR=0.02; 95% CI: 0.00–0.49) and smoking status (OR=0.66; 95% CI: 0.54–0.81, and AOR=0.001; 95% CI: 0.00–0.02).

## DISCUSSION

Despite the strong and consistent epidemiological evidence linking cigarette smoking to several CVDs and liver diseases^27^, the association between smoking intensity and changes in CVD risk factors remains unclear. Therefore, this study aimed to explore the possible effects of cigarette smoking on cardiometabolic risk in 160 healthy individuals. The present study found that changes in laboratory findings were associated with smoking intensity and were worse in heavy smokers than in non-smokers. We selected criteria, notably the vWF, hs-cTnI, liver function, lipid profiles, and blood pressure, based on their association with CVD pathogenesis. These parameters provide a complete understanding of the underlying mechanisms, including dyslipidemia, endothelial dysfunction, cardiac damage, and liver damage, which may contribute to the onset and progression of CVD in smokers.

The measurement of vWF permits the evaluation of endothelial function and thrombotic risk in patients with CVD. Wannamethee et al.^20^ argued that the early detection of endothelial damage is a useful step in diagnosing atherosclerosis. The assessment of vWF functional activity, a marker of endothelial damage, revealed significantly higher levels among smokers than non-smokers^20^. Although the mechanism by which smoking influences vWF release is not fully understood, it has been suggested that lipid peroxidase formed by O2 free-radicals and the effects of nicotine and CO2 contribute to the increase in vWF functional activity^28^.

Monitoring hs-cTnI levels enables the detection and evaluation of cardiac injury and provides vital diagnostic and prognostic information for patients with CVD. In the current study, hs-cTnI levels were significantly higher among smokers compared to non-smokers. Our findings are in contrast to those of a recent study by Skranes et al.^29^, which reported lower hs-cTnI concentrations in smokers and an inverse relationship between smoking and hs-cTnI levels, excluding cardiac damage among smokers. The authors explained that younger smokers had better cardiac risk profiles than non-smokers in their study. This discrepancy between studies may be attributed to differences in sample characteristics, sampling techniques, or the types of cigarettes smoked by the participants. However, our study’s elevated hs-cTnI levels among heavy smokers suggested early injury to the endothelium. The present study indicated that hs-cTnI levels were higher among heavy smokers who smoked >1 pack/day than among those who smoked ≤1 pack/day. However, the difference in mean hs-cTnI levels between the two groups was insignificant.

Cigarette smoke adversely affects various organs, including the liver and even organs not directly exposed to smoke. Additionally, the liver is involved in the processing and elimination of toxins from the body. The present study demonstrated that heavy smokers had significantly lower serum albumin levels, indicating potential impairment in liver function.

Furthermore, this study demonstrated that smokers had considerably lower overall mean blood bilirubin levels than non-smokers. While Alsalhen and Abdalsalam^30^ found a similar link which they attributed to the positive association between cigarette smoking and hemoglobin levels, our data indicate that the association between bilirubin concentration and smoking is independent of hemoglobin. The authors attributed the low blood bilirubin levels to higher quantities of free radicals, a direct outcome of cigarette smoking^30^.

In the present study, heavy smokers had a higher prevalence of dyslipidemia than moderate and non-heavy smokers. This observation is consistent with that of a previous study that showed an association between smoking and dyslipidemia, specifically with increased concentrations of triglycerides, LDL, and HDL^31^. Gastaldelli et al.^32^ supported these associations and further explained that nicotine promotes lipolysis, leading to an increased release of free fatty acids and the production of pro-atherosclerotic LDL. These associations were confirmed by the fact that quitting was associated with improved lipid metabolism, increased HDL levels, and decreased LDL concentrations, typically observed after a short period of abstinence. Additionally, oxidative stress and platelet aggregation caused by carbon monoxide and other oxidant gases produced during smoking contribute to dyslipidemia^32^.

The present study showed a significant impact of smoking on both BMI and the waist-to-hip ratio. Graff-Iversen et al.^33^ revealed a positive association between current smoking and the waist-to-hip ratio and concluded that smoking enhances abdominal obesity as an unhealthy outcome. Furthermore, Morris et al.^34^ used a Mendelian randomization approach to determine the causal effect of tobacco smoking on abdominal fat accumulation. Moreover, to exclude the possibility of confounders, logistic regression analysis revealed a significant odds ratio between smoking and obesity, with smokers having more than three times higher risk of being obese/overweight than non-smokers.

Our study showed that smokers had significantly higher systolic and diastolic blood pressure than non-smokers. Moreover, the number of daily cigarettes was positively and significantly associated with the participant’s systolic and diastolic blood pressures. Similarly, in a study by Mann et al.^35^, 24-hour ambulatory blood pressure monitoring revealed that smokers maintained a higher mean day-time ambulatory systolic blood pressure than non-smokers. Primatesta et al.^36^ stressed that smoking causes an acute increase in blood pressure and is associated with malignant hypertension. The high blood pressure among smokers could be explained by nicotine acting as an adrenergic agonist, mediating local and systemic catecholamine release, and possibly vasopressin release.

### Strengths and limitations

The present study has several limitations, including self-reported smoking habits, non-probability sampling, the possibility of selection and/or recall bias, residual confounding, the cross-sectional study design, which is good for hypothesis generation rather than hypothesis testing, and the limited sample size. Therefore, the generalizability of our findings to other countries is limited. However, this study has several strengths. This study provides valuable insights into the effects of smoking on various laboratory findings, including lipid profiles, vWF functional activity, serum albumin levels, troponin I levels, and liver function.

## CONCLUSIONS

Based on the findings of the present study, it can be concluded that heavy smokers and those who smoked for longer durations have significantly worse lipid profiles and higher vWF functional activities than other participants. Moreover, hs-cTnI levels, blood pressure, BMI, and waist-to-hip ratio were higher in smokers than in non-smokers, possibly reflecting a higher susceptibility to CVD.

Therefore, smoking cessation programs should be implemented, and comprehensive health promotion programs should be widely applied. Primary healthcare providers can actively participate in reducing smoking-induced consequences related to cardiometabolic diseases by initiating innovative health promotion programs. Further prospective studies with larger sample sizes are essential to supplement the results of this study.

## Supplementary Material



## Data Availability

The data supporting this research can be found in the Supplementary file.
