# Corrigendum: Association of cigarette smoking with cardiometabolic risk factors: A cross-sectional study

**DOI:** 10.18332/tid/196812

**Published:** 2024-12-12

**Authors:** Samar Sultan, Fouzy Lesloom

**Affiliations:** 1 Medical Laboratory Sciences, Faculty of Applied Medical Sciences, King Abdulaziz University, Jeddah, Saudi Arabia; 2 Regenerative Medicine Unit, King Fahad Medical Research Centre, King Abdulaziz University, Jeddah, Saudi Arabia

**Keywords:** smoking, cardiometabolic risk factors, liver function tests, high-sensitivity cardiac troponin I

Corrigendum on:

Association of cigarette smoking with cardiometabolic risk factors: A cross-sectional study

By Samar Sultan, Fouzy Lesloom

Tobacco Induced Diseases, Volume 22, Issue July, Pages 1-10,

Publish date: 26 July 2024

DOI: https://doi.org/10.18332/tid/191246

The CONFLICTS OF INTEREST statement in the originally published version of the article, is now corrected as follows:

‘The authors have completed and submitted the ICMJE Form for Disclosure of Potential Conflicts of Interest. The authors declare that they have no competing interests, financial or otherwise, related to the current work. All authors report that in the past 36 months, they received support from institutional funds projects from the Ministry of Education and King Abdulaziz University, Jeddah, Saudi Arabia (Grant number: IFPRC-126-290-2020).’

Additionally, in the originally published version of the article, the FUNDING statement was not provided. The statement is now corrected as follows:

‘This research work was supported by institutional funds projects from the Ministry of Education and King Abdulaziz University, Jeddah, Saudi Arabia (Grant number: IFPRC-126-290-2020).’

The mentioned changes have also been corrected online.

