# Supplementary material for: Association of cigarette smoking with cardiometabolic risk factors: A cross-sectional study
Source: Tob Induc Dis. 2024 Jul 26;22:10.18332/tid/191246. doi: 10.18332/tid/191246 (PMC11274995; doi:10.18332/tid/191246)
Supplement: Supplementary file 1 [file TID-22-136-s1.pdf]

Association of Cigarette Smoking with Cardiometabolic Risk Factors: A Cross-Sectional Study

Supplementary tables

Table S1 Results of cardiometabolic factors according to participants' smoking intensity (pack/day) (n=100)

Parameters	≤1 pack/day (n=80)		>1 pack/day (n=20)		P value
	Mean	SD	Mean	SD	
Triglycerides	149.79	11.50	144.33	17.34	0.248
Total cholesterol	198.39	11.79	209.00	16.78	0.014†
LDL-cholesterol	113.36	17.99	127.58	15.17	0.011†
HDL cholesterol	49.04	7.20	53.92	11.37	0.109
Non-HDL level	118.61	16.23	135.92	27.29	0.017†
ALT (U/L)	29.57	2.33	31.00	1.76	0.065
Albumin	4.68	0.29	4.41	0.39	0.021†
Total bilirubin	0.95	0.05	0.92	0.05	0.360
Fasting blood glucose	88.50	5.99	91.08	8.16	0.270
Fibrinogen level	3.49	0.56	3.76	7.12	0.433
vWF functional activity	57.25	7.12	81.42	28.51	<0.001†
Cardiac troponin I (ng/mL)	0.037	0.008	0.041	0.008	0.192
Systolic blood pressure	134.1	9.2	139.8	10.4	0.004†
Diastolic blood pressure	93.9	6.5	97.9	7.3	0.004†
Waist-to-hip ratio (males)	0.97	0.11	1.05	0.13	0.020†
Waist-to-hip ratio (females)	0.90	0.07	0.89	0.01	0.672

† Independent variable t-test (statistically significant)

Table S2 Crude odds ratio (OR) and adjusted odds ratio (AOR) of study variables related to obesity/overweight (BMI >25 kg/m^2) among all participants (n=160)

Independent Variables	OR	OR 95% CI		AOR	AOR 95% CI †	
		Lower	Upper		Lower	Upper
Age groups	1.559	0.773	3.145	1.494	0.287	7.783
Gender	0.914	0.474	1.762	4.132	0.546	31.278
ALT Level	0.855	0.438	1.670	3.027	0.381	24.048
Triglyceride Level	0.688	0.360	1.314	0.989	0.188	5.217
Total Cholesterol Level	2.359	1.220	4.561	0.108	0.010	1.118
Fasting blood glucose	0.663	0.541	0.812	0.016	0.001	0.492
LDL Level	1.310	0.541	3.171	1.071	0.123	9.353
Total bilirubin Grade	0.216	0.077	0.606	3.073	0.326	28.925
HDL Grade	4.197	1.185	14.861	1.274	0.099	16.374
Fibrinogen level	1.857	0.692	4.981	0.130	0.013	1.269
Smoking Status	0.161	0.088	0.295	0.001	0.000	0.015
Constant	--			1423.9	--	

† Binary logistic regression analysis

PART A

Section 1: Personal characteristics

- Age: years
- Gender: (1) Male (2) Female
- Residence: (1) Urban (2) Rural
- Education: (1) Illiterate (2) Primary (3) Intermediate (4) Secondary (5) University
- Marital status: (1) Single (2) Married
- If married, does your spouse smoke? (1) Yes(2) No
- Weight: kg
- Height: cm
- Body mass index: kg/m^2
- Waist circumference: cm

Section 2: Smoking pattern

- How old were you when you tried your first cigarette? Years
- When was the last time you smoked?

  (1) Never (go to next section) (2) 1-30 days (3) 1-6 months

  (4) >6 months
- Types of smoking: (1) Cigarettes (2) Shisha (3) Others
- How many cigarettes do you smoke daily? (0) I do not smoke

  (1)≤ 10 (2) 11-20 (3) 21-40 (4) >40
- Do you find it difficult to refrain from smoking in the “No Smoking” places?

  (1) Yes (2) No

Section (C): Assessment of cardiometabolic risk

- Fasting blood glucose
- Lipid profile
- High sensitive troponin I
- Von Willebrand factor
- ALT
- Albumin
- Total bilirubin

©2024 Sultan S. and Lesloom F.
